# School-based mental health promotion in children and adolescents with *StresSOS* using online or face-to-face interventions: study protocol for a randomized controlled trial within the ProHEAD Consortium

**DOI:** 10.1186/s13063-018-3159-5

**Published:** 2019-01-18

**Authors:** Heike Eschenbeck, Laya Lehner, Hanna Hofmann, Stephanie Bauer, Katja Becker, Silke Diestelkamp, Michael Kaess, Markus Moessner, Christine Rummel-Kluge, Hans-Joachim Salize, the ProHEAD Consortium, Michael Kaess, Stephanie Bauer, Rainer Thomasius, Christine Rummel-Kluge, Heike Eschenbeck, Hans-Joachim Salize, Katja Becker, Katja Bertsch, Sally Bilic, Romuald Brunner, Johannes Feldhege, Christina Gallinat, Sabine C. Herpertz, Julian Koenig, Sophia Lustig, Markus Moessner, Fikret Özer, Peter Parzer, Franz Resch, Sabrina Ritter, Jens Spinner, Silke Diestelkamp, Kristina Wille, Sabrina Baldofski, Elisabeth Kohls, Lina-Jolien Peter, Vera Gillé, Hanna Hofmann, Laya Lehner, Elke Voss, Jens Pfeiffer, Alisa Samel

**Affiliations:** 1 Department of Psychology, University of Education Schwäbisch Gmünd, Oberbettringer Str. 200, 73525 Schwäbisch Gmünd, Germany; 2 Center for Psychotherapy Research, University Hospital Heidelberg, Bergheimerstr. 54, 69115 Heidelberg, Germany; 3 Department of Child and Adolescent Psychiatry, Psychosomatics and Psychotherapy, University Hospital of Marburg and Philipps-University Marburg, Hans-Sachs-Str. 6, 35039 Marburg, Germany; 4 Marburg Center for Mind, Brain and Behavior (MCMBB), Philipps-University Marburg, 35043 Marburg, Germany; 5 University Hospital Hamburg-Eppendorf, German Center for Addiction Research in Childhood and Adolescence, Martinistr. 52, W29, 20246 Hamburg, Germany; 6 Section for Translational Psychobiology in Child and Adolescent Psychiatry, Department of Child and Adolescent Psychiatry, Centre for Psychosocial Medicine, University Hospital Heidelberg, Blumenstraße 8, 69115 Heidelberg, Germany; 7 University Hospital of Child and Adolescent Psychiatry and Psychotherapy, University of Bern, Stöckli, Bolligenstrasse 141c, 3000 Bern 60, Switzerland; 8 Department of Psychiatry and Psychotherapy, University Leipzig, Semmelweisstraße 10, 04103 Leipzig, Germany; 9 Mental Health Services Research Group, Central Institute of Mental Health, Medical Faculty Mannheim/Heidelberg University, J5, 68159 Mannheim, Germany

**Keywords:** Mental health promotion, Prevention, School, Stress, Coping, Mental health literacy, Online intervention, Adolescents, Randomized controlled trial, ProHEAD

## Abstract

**Background:**

Schools are an ideal setting in which to promote health. However, empirical data on the effectiveness of school-based mental health promotion programs are rare, and research on universal Internet-based prevention in schools is almost non-existent. Following the life skills approach, stress management training is an important component of health promotion. Mental health literacy is also associated with mental health status, and it facilitates formal help-seeking by children and adolescents (C&A). The main objectives of this study are (1) the development and evaluation of an Internet-based version of a universal school-based health promotion program called *StresSOS* and (2) demonstrating non-inferiority of the online setting compared to the face-to-face setting. *StresSOS* aims to improve stress management and mental health literacy in C&A.

**Methods/design:**

A school-based sample of 15,000 C&A (grades 6–13 and older than 12 years) will be recruited in five regions of Germany within the ProHEAD Consortium. Those with a screening result at baseline indicating no mental health problems will be invited to participate in a randomized controlled trial comparing *StresSOS* online to an active online control condition (Study A). In addition, 420 adolescents recruited as a separate school-based sample will participate in the *StresSOS* face-to-face intervention. Participants in both intervention groups (online or face-to-face) will receive the same eight treatment modules to allow for the comparison of both methods of delivery (Study B). The primary outcome is the number of C&A with symptoms of mental health problems at a 12 months follow-up. Secondary outcomes are related to stress/coping (i.e., knowledge, symptoms of stress, coping resources), mental health literacy (knowledge and attitudes toward mental disorders and help-seeking), program usage patterns, cost-effectiveness, and acceptability of the intervention.

**Discussion:**

This study represents the first adequately powered non-inferiority trial in the area of school-based mental health promotion. If online *StresSOS* proves efficacious and non-inferior to face-to-face delivery, this offers great potential for health promotion in youths, both in and outside the school environment.

**Trial registration:**

German Clinical Trials Register, DRKS00014693. Registered on 14 May 2018.

**Electronic supplementary material:**

The online version of this article (10.1186/s13063-018-3159-5) contains supplementary material, which is available to authorized users.

## Background

Mental health problems in childhood and adolescence are key health issues (see, e.g., [1]). In children and adolescents (C&A) mental health problems present a burden for individuals, their families, and the social environment that may persist over the lifespan. Greater efforts for mental health promotion and prevention interventions are desperately needed. Schools are an ideal setting in which to promote health [2]. The school is the place where C&A spend a lot of their time. It influences the cognitive, social, and emotional development of C&A for years and contributes to the stabilization of behavioral habits (including health behaviors). School is the place where most young people can be reached, and it provides access to cohorts of C&A as a whole within a region. It establishes an organizational framework for group-related interventions and their evaluations [3]. Yet studies documenting health promotion activities at schools (e.g., [4, 5]) that focus on the promotion of mental health and wellbeing are the exception, and most evaluated interventions have been developed and implemented in the USA [6].

School-based health promotion or prevention programs often follow a universal approach and are delivered to all individuals of a cohort. Meta-analyses have identified a range of positive small to moderate effects of school-based mental health promotion with large impact [6]. The “Life Skills” training [7, 8] is a prototypical health promotion approach that focuses on enhancing social and personal skills to enable individuals to deal effectively with the demands and challenges of everyday life. Core life skills include communication, decision-making and problem-solving, and coping with emotions and stress (see the World Health Organization (WHO) definition [7]). How individuals cope with stressful situations strongly influences the impact stress has on adjustment and health [9, 10]. Stress involves a person’s response pattern to particular events (i.e., stressors) that disturb the individual’s equilibrium and tax or exceed his or her ability to cope. Thus, stressors and coping are important mental health factors. Transactional definitions [10] point to the degree to which situations are perceived as taxing or exceeding resources and endangering a person’s wellbeing, not solely to the situation itself. Consequently, an individual’s appraisal of events or situations (e.g., demands of school; conflicts among family, friends, or peers) and of his/her resources is important. Coping refers to changing cognitive and behavioral efforts to manage specific demands that are appraised as taxing or exceeding one’s resources [10]. In past years, school-based stress prevention training programs (mostly face-to-face) have been developed for C&A (see, e.g., [11–13]). Programs typically focus on knowledge about stress and strategies to deal with it (e.g., problem-solving, alternative/positive thinking strategies, relaxation techniques, social support seeking). Evidence supports benefits from the intervention (e.g., increased knowledge, more adaptive coping). In their meta-analysis on school-based stress management programs (19 studies included), Kraag et al. [14] tentatively conclude that school programs targeting stress/coping are effective in reducing stress symptoms and enhancing coping skills (see also [6, 15]). Yet, future research should be methodologically stronger (e.g., with regard to outcome measures or follow-up data).

Internet-based interventions hold many advantages; for example, they can save resources, such as teachers’ classroom time or researchers for program delivery, and enhance reach. They are easily accessible for C&A, who spend a lot of time using electronic media [16]. Among others, one function of electronic media use, including the Internet, is to help a person cope with stress [17]. Reviewing the effectiveness of online mental health promotion and prevention interventions for C&A, Clarke et al. [18] identified only two online stress management interventions [19, 20]. Both types of training correspond in content with face-to-face stress prevention programs (i.e., providing knowledge about stress and strategies to deal with stress). The authors reported improvements in knowledge about stress and coping and showed first indications for more adaptive coping strategies.

With the aim of coping with stressors or strains in the future, mental health literacy (MHL) is also an important concept. Adolescence is a period of significant risk for mental disorders [21]. However, if mentally ill, a large proportion of adolescents fail to obtain the needed mental health care. A recent review (based on 22 studies [22]) of perceived barriers and facilitators for mental health help-seeking in young people showed that perceived stigma and embarrassment, problems recognizing symptoms, and a preference for self-reliance were the most important barriers. However, perceived positive past experiences, social support, and encouragement from others were identified as facilitators of the help-seeking process. The review concluded that strategies for improving help-seeking in C&A should focus on improving MHL and reducing stigma. MHL has been defined as “knowledge and beliefs about mental disorders which aid their recognition, management or prevention” [23]. Following Jorm [23], MHL encompasses knowledge that is linked to the possibility of action to benefit one’s own mental health or that of others including knowledge of how to prevent mental disorders, recognition of when a disorder is developing, knowledge of help-seeking options and treatments available, knowledge of effective self-help strategies for milder problems, and first aid skills to support others who are developing a mental disorder or are in a mental health crisis. School-based MHL programs address basic concepts of mental health, provide strategies for help-seeking, or include activities for stigma reduction toward mental illness. However, programs are heterogeneous with regard to the focus of the interventions, duration, and dose (for a review, see [24]). For example, the HeadStrong intervention [25] is a universal, curriculum-based program on MHL, stigma, help-seeking, psychological distress, and suicidal ideation. Classroom activities are delivered by teachers over a period of 5–8 weeks in five modules (e.g., “Mood and mental wellbeing”, “Reaching out – helping others”). *The Guide* resource (teenmentalhealth.org) consists of six modules covering stigma and mental illness, understanding mental health and mental illness, information on specific mental illnesses, experiences of mental illness, help-seeking, and the importance of mental health [26]. Reviewing the effectiveness of school-based MHL programs (Cochrane criteria applied, 27 studies included), Wei and colleagues [24] state that even if most studies report improved knowledge (12 of 15 studies), changes in stigmatizing attitudes (14 of 21 studies), and/or enhanced help-seeking behavior (8 studies, of which 5 studies investigated attitudes toward help-seeking; results were mixed for different sources), the quality of the evidence is low. Methodological weaknesses include, but are not limited to, the lack of randomized controlled trials (RCTs), missing control for confounders (e.g., age, gender, mental health status), and the lack of validated measures. In addition, former reviews on school-based MHL interventions [27, 28] primarily criticize the quality of research designs (e.g., small sample sizes, lack of follow-ups). Regarding Internet resources, there are various websites to improve youth MHL skills (e.g., mindcheck.ca, foundrybc.ca; see [29]). Preliminary pilot data support the idea that brief Internet-based MHL interventions improve knowledge and help-seeking attitudes [30]. The topics covered were, for example, depression, anxiety, and suicide.

To summarize, in terms of resource promotion and empowerment among C&A, both coping skills and MHL are powerful resources. C&A benefit from a broad spectrum of coping strategies to alter a specific stressful situation (e.g., problem-solving, instrumental support seeking) or adjust to the stressor (e.g., emotion regulation, emotional support seeking) to enhance individual strengths to manage challenges and demands. MHL enables self-help strategies for milder problems and, for example, first aid skills to support others. Preliminary evidence on universal school programs targeting stress/coping or MHL (mostly face-to-face) tentatively supports benefits for mental health in C&A. However, existing reviews on the effectiveness of stress/coping or MHL preventions clearly document methodological weaknesses and conclude that there is a need for more research targeting stress/coping and MHL utilizing sound designs (e.g., RCTs with pre-test, post-test, follow-up, adequate sample size, and outcome measures). Advantages of Internet-based programs are evident (e.g., personnel and time resources, accessibility, reach, attractiveness, target-group specificity); however, research is still in the early stages.

### Objectives

The aim of the study is the development, implementation, and evaluation of a health promotion program named *StresSOS* addressing stress management and MHL. It is planned to investigate the efficacy of Internet-delivered *StresSOS* compared to an active online control condition (Study A) and non-inferiority of *StresSOS* online delivery to *StresSOS* face-to-face delivery (Study B) with regard to a reduction of new incidence of mental health problems in C&A. The primary hypothesis is that significantly fewer C&A in the *StresSOS* group will transit from the “healthy” group to the “high-risk” or the “mental health problems” group at 12 months follow-up, when compared to a control group receiving an online program on healthy nutrition. Further, we will explore for whom the program is most effective (e.g., child demographics). The programs’ acceptability will be analyzed to identify factors supporting or hindering the implementation of the Internet-based or the face-to-face prevention program at schools. In addition, data will be collected on the cost of interventions and compared to the study outcomes to determine the cost-effectiveness ratio of interventions.

## Methods/design

### Design

**Fig. 1:**
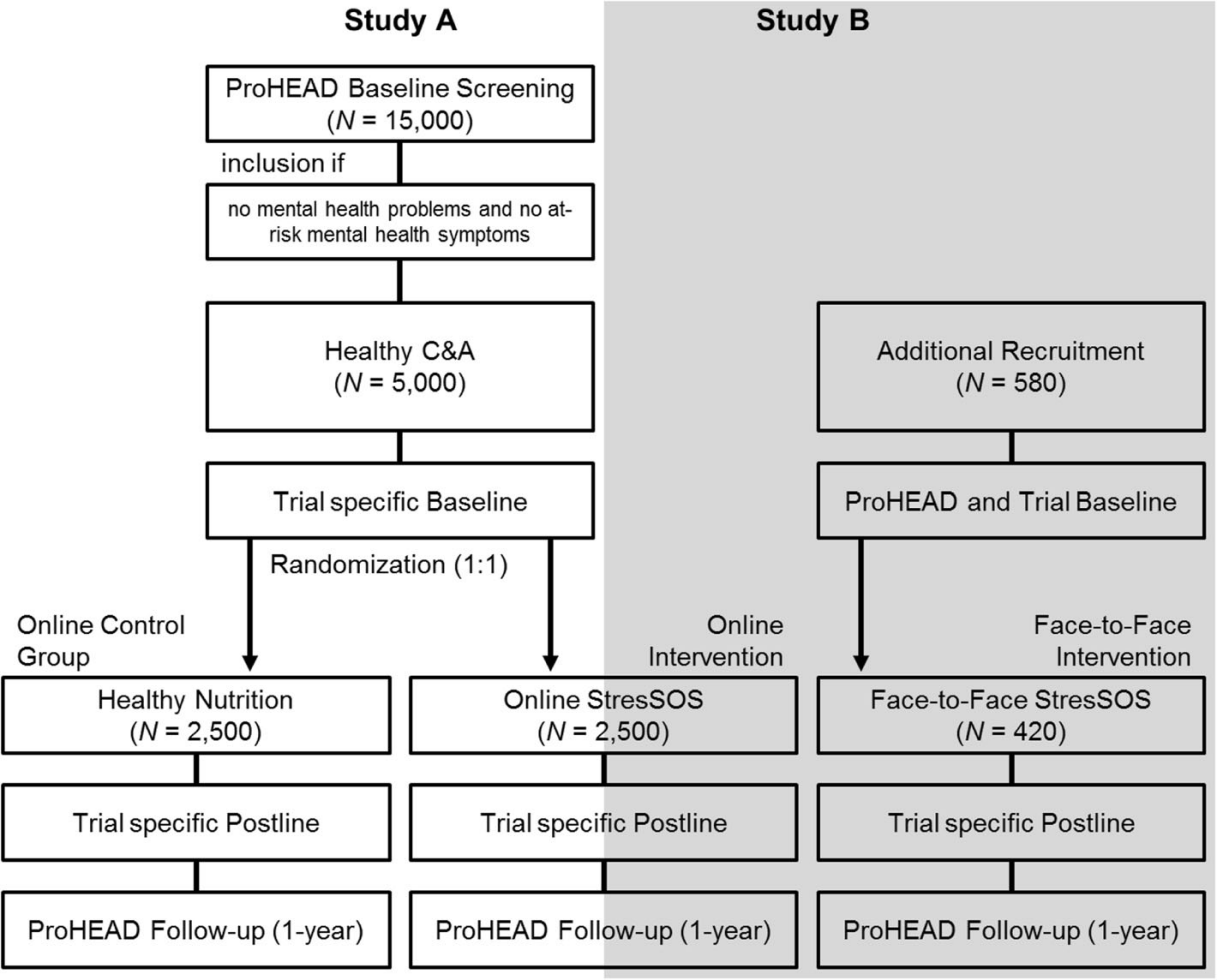
StresSOS trial intervention scheme and trial flow. Note: Participants will complete the ProHEAD baseline screening and 1 and 2 year follow-up assessments within the ProHEAD Consortium. In addition, assessments within the *StresSOS* trial will be conducted at baseline and post-intervention (8 weeks after onset of intervention)

ProHEAD is a longitudinal study with three school-based screenings at intervals of 12 months assessing mental health problems in C&A. Further, it is an interventional study, allocating C&A to different programs tailored to their individual needs and evaluating the efficacy and cost-effectiveness in a randomized controlled design (see also https://www.prohead.de/). The *StresSOS* trial is an RCT within the ProHEAD Consortium. Healthy adolescents will take part in either a face-to-face *StresSOS* intervention at school, in a *StresSOS* online intervention at home, or in an online control group. Participants in both intervention groups (online or face-to-face) will receive the same treatment modules. The efficacy of the online program will be investigated as compared to the control condition (Study A) and non-inferiority of online delivery compared to face-to-face delivery (Study B); see Fig. 1. Additional file 1 provides the completed Standard Protocol Items: Recommendations for Interventional Trials (SPIRIT) checklist.

### Recruitment

Participants for the Internet-delivered *StresSOS* (and the corresponding online control group) will be recruited within the ProHEAD Consortium via schools in five regions of Germany (Hamburg, Heidelberg, Leipzig, Marburg, Schwäbisch Gmünd). Details on the recruitment can be found in Kaess et al. [31]. Participants for the face-to-face *StresSOS* program will be recruited as an additional sample via schools in the region of Schwäbisch Gmünd, Germany.

### Enrollment and randomization

Permission to contact schools within the regional districts of all five recruiting study sites (Hamburg, Heidelberg, Leipzig, Marburg, Schwäbisch Gmünd) will be requested from federal authorities. A total list of schools in the regional districts will be acquired. Eligible schools will be contacted and informed, in random order, about the possibility to participate, until the prospected sample size is reached (in total *N* = 15,000 C&A). Following the baseline screening, healthy C&A (not fulfilling allocation criteria for any other ProHEAD program; see [31–34]) will be invited to participate in the *StresSOS* online program. C&A will receive an email including information about the trial and access details for the baseline assessment within the *StresSOS* trial. After completing this assessment, participants will be randomized with equal probability (in a 1:1 ratio) to the online *StresSOS* program and the online control group (online program on healthy nutrition). The allocation will be randomized, stratified for gender and school type. Randomization will be conducted externally and will follow a permutated block design with variable, randomly arranged block sizes. Following randomization, participants will be contacted informing them about their allocation to the respective intervention group and will receive access details for the specific online program.

In the face-to-face *StresSOS* program, the entire class will be invited to participate in the trial. Parallel to the online trial, eligible schools will be contacted and informed in random order about the possibility of participating in the program. Blinding is not possible. Participating C&A and experts are not unaware of the interventional procedure. However, assessments are based on self-report and take place within the school-based screening, which helps to avoid biased assessment of results.

### Inclusion and exclusion criteria

C&A from participating schools who are in grades 6–13 and older than 12 years and who provide both written informed consent forms (children, adolescents and parents) are invited to participate in the ProHEAD baseline screening. Following the baseline screening, C&A will be identified as currently either “healthy”, “high-risk” (including sub-threshold mental health problems and diverse health-risk behaviors), or having “mental health problems” (which includes clinically relevant levels of psychopathology and/or indicators of threat to self and others). Based on these screening profiles, C&A will be allocated to one of the five ProHEAD online trials: increasing help-seeking in C&A with general mental health problems [31]; reducing eating disorder symptoms [32]; reducing at-risk alcohol use [33]; reducing depressive symptoms [34]; promoting mental health in healthy C&A (this *StresSOS* RCT). That is, healthy C&A without mental health problems and without at-risk mental health symptoms as assessed in the school-based ProHEAD screening not fulfilling allocation criteria for the other four ProHEAD online programs [31–34] are invited to participate in the *StreSOS* trial. Further inclusion criteria are sufficient German language skills and Internet access. Criteria for the allocation of participants to the five individual ProHEAD trials (i.e., this *StresSOS* RCT and the other four programs [31–34]) are based on the latest scientific evidence. However, this is the first time that the overall algorithm will be applied on a consortium-wide basis simultaneously screening for various mental health problems. Therefore, an intermediate data analysis will be conducted following completion of 10% of the screening assessments (*N* = 1500 C&A) in order to determine the actual allocation ratio to the five ProHEAD trials and to adjust the screening algorithm if necessary.

### Participants’ incentive

Participants receive no direct financial compensation for participating in the school-based assessments. Among all participating students, a draw will be conducted, awarding vouchers for online shopping portals (20€ in value) to 5% of the participants.

### Intervention

All interventions (online and face-to-face) comprise eight weekly sessions. Each session provides knowledge and information referring to the session’s main issue, reinforces content by illustrating particular examples, tests participants in a playful manner (e.g., with quizzes and exercises on the session’s topic), and fosters transfer to daily life with a weekly homework assignment aiming to increase self-management skills.

### *StresSOS* (online or face-to-face)

The *StresSOS* program focuses on stress/coping and MHL. It teaches coping skills (e.g., problem-solving, cognitive reconstruction, relaxation techniques, seeking support), the connection between thoughts, feelings, and behaviors, and concepts about mental health/illness and help-seeking. See Table 1 for a content overview of the *StresSOS* sessions (online and face-to-face). The *StresSOS* program is based on the life skills approach, especially stress prevention programs (e.g., [11, 12]) and MHL programs (e.g., [25, 26]).

**Table 1:** *StresSOS* module content

Module number and content	Content overview
1. The basics of stress and wellbeing	What is stress? How does stress affect the body? How does stress occur? Is stress a disease?
2. Managing stress: problem-solving	When do I get stressed? What can I do if I find myself in a stressful situation? I have a problem — how can I solve it?
3. Helpful thoughts	Stress occurs in the head — why thoughts play a role. Relationship between thoughts, feelings, and behavior. My helpful thoughts and feelings — my hindering thoughts and feelings. How can I change my thoughts and expectations?
4. Time to chill out and relax	What happens in the body during relaxation? What possibilities for balance and recreation are there? What are relaxation methods?
5. Upward spirals of positive emotions	Spiral of emotions — up or down? What is the spiral of positive emotions? What makes me feel good? How can I influence my emotions? What do positive feelings and stress management have in common?
6. Seek support and talk	What is social support and why is it so important? Social support — a give and take. Whom can I contact if I feel I can’t cope on my own? How can I make social contacts? How do I express socially competent and self-confident behavior?
7. Mental health and mental illness	What is mental health? What are risk factors and protective factors? Stress and mental illness — what’s the difference? Mental illness in adolescents — is this an issue at all? How can I help others?
8. A glimpse into the future — finding one’s own goals	Finding the appropriate goals. Why are goals important? What do goals have to do with stress and wellbeing? Why can thinking about goals be helpful?

The *StresSOS* face-to-face program is a classroom-based intervention, administered by experts (advanced students, mental health professionals). One weekly session lasts 90 min. A variety of teaching methods will be included (e.g., presentations, group discussion, group activities, role play, video presentations, games, quizzes, answer sheets, homework). The *StresSOS* online program will be completed by C&A autonomously (e.g., from their computer at home). A weekly session takes approximately 20–90 min according to individual needs and preferences. The program contains information and psycho-educational materials, interactive online training, quizzes, and exercises on the session’s topic. An individualized monitoring and feedback module is implemented. Once a week participants will receive an email that reminds them about the upcoming session. Via a link in the email they can access an online monitoring questionnaire, which asks them whether they have completed their “homework” (“Your turn! Give it a try”) and whether they liked it. The following individualized feedback will reinforce positive behaviors and offer suggestions and recommendations in case C&A did not complete their homework or did not experience the task as helpful or good. A group chat moderated by an expert takes place once a week. C&A are invited to join the chat on a voluntary basis and have the opportunity to ask questions of the expert or discuss their experiences within the program with their peers. Finally, the program contains a news blog. Moderators frequently upload information to the news blog and provide up-to-date news about the topic.

### Active online control condition (program for healthy nutrition)

The online control intervention focuses on healthy nutrition (a topic that has no direct link to the intervention addressing stress management and MHL). It can be used according to individual needs and preferences and aims to educate C&A on the following topics about nutrition in order to promote healthy eating: (1) Basics about healthy nutrition, nutrients and energy; (2) Fruit and vegetables; (3) Breads, cereals, and potatoes; (4) Milk, dairy products, fish, meat, sausages, and eggs; (5) Healthy drinks; (6) Many different foods, enjoy the diversity of foods available; (7) Healthy breakfast and healthy snacks; (8) Eating when you are out. All contents are based on recommendations provided by the German Society for Nutrition [35]. The technical functions are parallel to the online intervention *StresSOS* with the exception that there will be no group chat.

### Sample size calculation

In the control condition, 30% transitions from “healthy” to “at-risk” are expected. Further, it is anticipated that participation in *StresSOS* (online or face-to-face) will prevent 50% of these transitions, i.e., reduce the transitions to 15%. The sample size calculation is primarily based on the non-inferiority trial (Study B). Assuming equal efficacy for online and face-to-face delivery of *StresSOS* (i.e., 15% transition to “at-risk”), a non-inferiority margin δ = 5%, and an allocation ratio of 5:1, 2920 participants (2500 in the online and 420 in the face-to-face condition) need to be included in Study B to test non-inferiority with 80% power at α = 5% (Z-test). For Study A, the proposed sample size will allow one to assess the assumed group differences precisely (95% confidence interval (CI) +/− 2.3%); i.e., it allows one to test clinical significance of the efficacy of online *StresSOS* (between-group difference > 10%) with more than 95% power. The sample size may be larger, as all C&A who do not meet the inclusion criteria of the other four ProHEAD trials for C&A with (sub-threshold) mental health problems [31–34] are included. Even if the proposed sample size is reached, the program will still be offered to “healthy” C&A.

Based on previous experiences in school-based settings, there may be an expected loss to follow-up rate of 15% for the face-to-face sample [36] and 25% for the program-specific assessments of the online sample. Based on the literature [19] and the experiences within the consortium, the expectation is that about 33% of the participants in the online *StresSOS* condition will be compliant and work through the modules as expected, 33% will utilize about half of the modules, and 33% will log in only once.

### Measures

#### Primary outcome measures

The primary outcome is the number of C&A with transition from the “healthy” group to the “high-risk” or the “mental health problems” group at 12 months follow-up, i.e., the proportion of C&A fulfilling any of the criteria for at-risk C&A or for C&A with mental health problems outlined in the ProHEAD trials on general mental health problems, eating disorder symptoms, at-risk alcohol use, and depressive symptoms (see [31–34] in this issue). Data will be assessed in the school-based follow-ups at 1 and 2 years post-enrollment within the ProHEAD Consortium.

The measures include the following instruments. The *Strengths and Difficulties Questionnaire* (SDQ) [37] is a screening questionnaire for psychosocial problems in C&A. It comprises four sub-scales (emotional, conduct, hyperactivity, and peer problems), each scored on a scale from 0 to 10. Instruments specific for the assessment of eating disorder symptomatology include the *Short Evaluation of Eating Disorders* (SEED) [38] and the *Weight Concerns Scale* (WCS) [39]. The SEED assesses the three main symptoms for anorexia (degree of underweight, fear of weight gain, and distortion of body perception) and bulimia (amount of binge eating, amount of compensatory behavior, and over-concern with body shape and weight). The WCS uses five items to assess concerns with weight associated with body image in women. Instruments specific for the assessment of risky alcohol use and alcohol misuse include the *Car, Relax, Alone, Forget, Friends, Trouble* questionnaire (CRAFFT-d) [40] and the *Alcohol Use Disorders Identification Test* (AUDIT) [41]. The CRAFFT-d is a behavioral health screening tool for at-risk alcohol use. The AUDIT is a screening tool developed by the WHO to assess alcohol consumption, drinking behaviors, and alcohol-related problems. As the main instrument for the assessment of depressive symptoms, the *Patient Health Questionnaire-9 modified for Adolescents* (PHQ-A) [42] is used. The PHQ-A is a nine-item self-report instrument assessing the presence and severity of depressive symptoms based on the diagnostic criteria for depression according to the Diagnostic and Statistical Manual of Mental Disorders, fourth edition (DSM-IV) [43].

#### Secondary outcome measures

In addition, assessments will be performed related to stress/coping (i.e., knowledge, symptoms of stress, coping resources), self-esteem, MHL (knowledge and attitudes about mental disorders and help-seeking), program usage patterns, cost of programs, and acceptance of the intervention. Data will be assessed in the school-based follow-ups at 1 and 2 years post-enrollment within the ProHEAD Consortium, except for the assessments related to knowledge, self-esteem, symptoms of stress, and coping resources, which will take place at the trial-specific post-line.

Knowledge about stress/coping and mental health will be assessed with a multiple-choice questionnaire (pretested in a pilot study with adolescents) measuring knowledge about stress (stressors, stress response, stress appraisals), different coping strategies and their implications (problem-solving, cognitive reconstruction, relaxation, seeking support), mental health/illness, and help-seeking. Symptoms of stress and coping strategies will be assessed using the *Stress and Coping Questionnaire for Children and Adolescents* (SSKJ 3–8 R) [44]. The physical symptoms scale consists of six items (e.g., headache, stomach ache; referring to the last week). With regard to coping, only the items related to coping with a social peer problem situation will be used with five-item sub-scales related to seeking social support, problem-solving, avoidant coping, and electronic media use. To assess self-esteem, the *Inventory of Self-Esteem for Children and Adolescents* (SEKJ) [45] will be used, applying the scales height and stability of self-esteem.

Three instruments are used to cover help-seeking intentions, actual help-seeking behavior, and attitudes toward help-seeking. The *General Help-Seeking Questionnaire* (GHSQ) [46] is a self-report measure of help-seeking intentions for selected mental health problems. The *Actual Help-Seeking Questionnaire* (AHSQ) [47] assesses actual help-seeking behavior by listing potential help sources and measuring whether or not help has been sought from the respective sources within a specified time period for a specified problem. It comprises three sub-scales: whether or not informal help has been sought, whether or not formal help has been sought, and whether no help has been sought. Further, the *Inventory of Attitudes Toward Seeking Mental Health Services* (IASMHS) [48] has three internally consistent factors: psychological openness, help-seeking propensity, and indifference to stigma. To assess barriers of help-seeking, the short form of the *Barriers to Adolescents Seeking Help Scale* (BASH-B) [49], including 11 items, will be used. Further, items from the *Questionnaire on Social Distance* [50], assessing stigma toward peers affected by mental health problems, will be used.

Participants will be asked to indicate their overall *program acceptance*; C&A provide an overall evaluation of the program and indicate whether they have learned something from it, and if they would recommend the program to friends or other adolescents. *Program user statistics* regarding the online intervention will be collected based on the system’s log files.

Health care utilization of participants will be collected by the *Mannheimer Modul Ressourcenverbrauch* (MRV), a scale that lists all possible health care services for a given study sample or risk group and reports the frequency of usage (e.g., visits, drug intake, hospital days) over a given period of time [51]. Similar scales are applied in international cost studies [52]. The MRV was modified and pretested for use in an adolescent population.

Health-related quality of life will be assessed using the *KIDSCREEN-10* [53]. The KIDSCREEN is an international cross-culturally comparable quality of life assessment instrument tailored for C&A aged 8 to 18 years.

#### Other measures

**Fig. 2:**
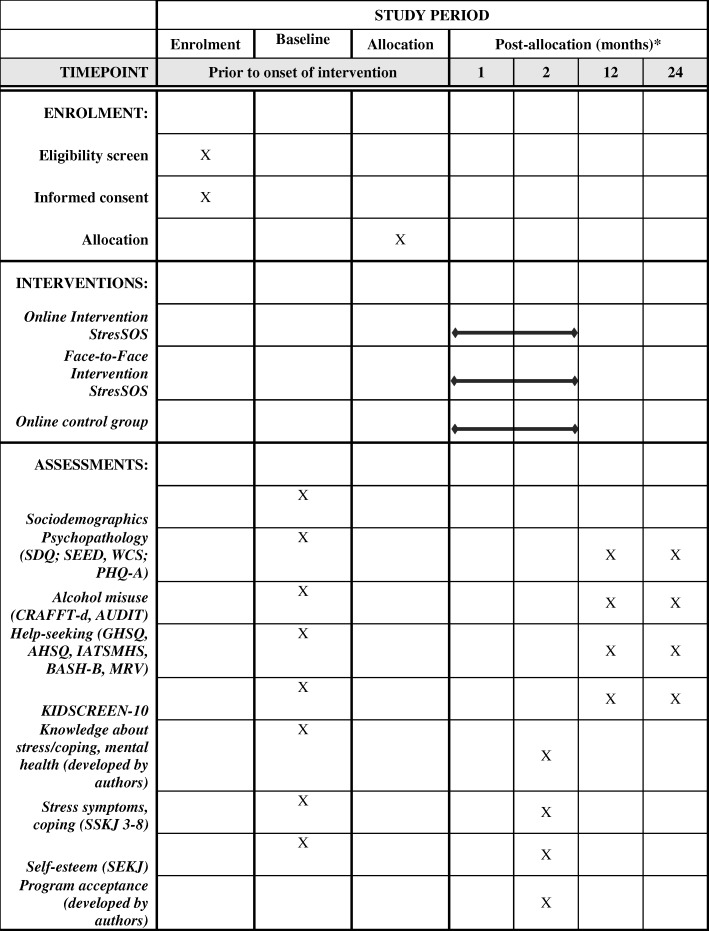
Schedule of enrollment, interventions, and assessments. Note: *SDQ* Strengths and Difficulties Questionnaire, *SEED* Short Evaluation of Eating Disorders, *WCS* Weight Concerns Scale, *PHQ-A* Patient Health Questionnaire-9 modified for Adolescents, *CRAFFT-d* Car, Relax, Alone, Forget, Friends, Trouble, *AUDIT* Alcohol Use Disorders Identification Test, *GHSQ* General Help-Seeking Questionnaire, *AHSQ* Actual Help-Seeking Questionnaire, *IASMHS* Inventory of Attitudes Towards Seeking Mental Health Services, *BASH-B* Barriers to Adolescents Seeking Help Scale, *MRV* Mannheimer Modul zum Ressourcenverbrauch, *KIDSCREEN-10* Health-related quality of life, *SSKJ 3–8 R* Stress and Coping Questionnaire for Children and Adolescents, *SEKJ* Inventory of Self-Esteem for Children and Adolescents. *Participants will complete the ProHEAD baseline screening and 1 and 2year follow-up assessments within the ProHEAD Consortium. In addition, assessments within the *StresSOS* trial will be conducted at baseline and post-intervention (8 weeks after onset of intervention)

Sociodemographics (i.e., gender, age, migrant status, socioeconomic status, etc.) will be assessed. For assessment of personality disorders, the *Standardized Assessment of Personality – Abbreviated Scale* (SAPAS) [54] will be used. The SAPAS is a self-report questionnaire consisting of eight items that screen for personality disorders. Figure 2 displays an overview of enrollment, interventions, and measures used as well as the corresponding time of assessment.

### Statistical analyses

#### Study A

Differences in the transition rate from the “healthy” group to the “high-risk” or the “mental health problems” group at 12 months follow-up will be tested with Fisher’s exact test (two- sided, α = 5%). The 95% CIs for the group difference will be calculated in order to test clinical significance (> 10% difference) of the online *StresSOS* efficacy. Analyses will be based on intention-to-treat (ITT) principles. Missing values will be imputed with the predictive mean matching method using the R package mice (Multivariate Imputation by Chained Equations [55]).

#### Study B

Non-inferiority [56] of online delivery of *StresSOS* will be tested against the null hypothesis H0: Difference (% transitions face-to-face – % transitions online) < 5% (Z-test, α = 5%). Non-inferiority is assumed if the upper limit of the 95% CI of the empirical group difference is less than 5% (CI inclusion approach). Propensity score weighting methods will be applied to control for potential confounders between the online and the face-to-face condition [57]. The covariates taken into account for the propensity score procedure will be, for example, psychological strain, coping skills, age, gender, and type of school. Tests will be based on ITT principles; missing values will be imputed.

#### Secondary outcomes

In order to test differences in secondary endpoints, effect sizes and CIs will be calculated. Repeated measures analysis of variance and covariance will be conducted to test effects on continuous secondary outcomes related to stress/coping (i.e., knowledge, symptoms of stress, coping resources) and MHL (knowledge and attitudes about mental disorders and help-seeking). In addition, cost-effectiveness analyses and cost-utility analyses of the study intervention will be conducted. Cost-effectiveness analyses include the calculation of the incremental cost-effectiveness ratio (ICER). The ICER indicates the additional cost for each additional (primary) outcome that has to be paid under routine care conditions. During these analyses, standard health economy techniques will be applied, such as bootstrapping techniques for estimating ICER variability, the calculation of cost-effectiveness acceptability curves (CEAC), and calculation of willingness-to-pay (WTP) criteria [52, 58]. In addition, a cost-utility study will be conducted that requires the transformation of longitudinal quality of life data (assessed with the KIDSCREEN-10) into preference measures, for the calculation of quality-adjusted life years (QALYs) lost or gained during follow-up in order to calculate costs per QALY as generated by the intervention.

### Organization, quality assurance, and data management

Online questionnaires will be used for pseudonymized data collection. To ensure confidentiality, a unique identifying code not linked to the real name will be assigned to each participant. Data quality will be ensured by conducting automatic validity and range checks at data entry. All study-related data will be kept in a secure environment, stored for 10 years on protected servers at the principal investigator’s institution with regular back-up procedures in place. Data handling and access will follow German and European Union legal regulations concerning data protection and data security. The Coordination Center for Clinical Trials (KKS) Heidelberg will monitor study-related procedures at the five recruiting centers. Specifically, the recruitment of schools within the target regions and the recruitment of students within these schools will be monitored in order to ensure adherence to the study manual and documentation guidelines as well as equivalent procedures at all sites. Furthermore, an independent data and safety monitoring board (DSMB) will assess the progress of the trial, data safety, and the clinical efficacy endpoints.

### Safety reporting

There is no obvious risk for participating C&A. All participants will receive information on where to seek help for mental health problems within the ProHEAD Consortium.

### Dissemination

Results will be presented at national and international conferences and published in peer-reviewed international journals. The Internet-based approach guarantees that the intervention can be sustained at a relatively low cost, providing support for a large population—on condition that the intervention proves efficacious.

## Discussion

Schools as a setting of health promotion show various benefits [3, 4]. Accordingly, health-promoting activities following the life skills approach, including stress prevention and the promotion of mental health, are present at schools (see, e.g., [11, 12, 25, 26]). Empirical data show that school-based (predominantly face-to-face) interventions are promising [14, 24]. However, dissemination requires trained professionals. Internet-based delivery would allow the provision of health promotion to schools independent of their geographic location. Further benefits of Internet-based programs to promote mental health in C&A are evident: programs are easily accessible, attractive, and resource-saving. Nevertheless, until now, research on universal school-based online prevention programs has been largely lacking (for exceptions, see [19, 20, 30]). The present study aims to overcome the limitations of existing research by investigating the efficacy of an Internet-based health promotion program for C&A and its non-inferiority compared to the face-to-face setting. Key questions are the potentially positive and negative effects of the program *StresSOS*, focusing on stress management and mental health with regard to the two different formats, face-to-face and Internet-based. This also leads to questions about differential effects which will be investigated in this ProHEAD trial. For example, which of the invited participants will complete the online *StresSOS* program? Are there different program effects related to gender, age, or socioeconomic status? To our knowledge, this is the first adequately powered non-inferiority trial in the area of school-based mental health promotion and the first trial that systematically investigates help-seeking behavior as an outcome variable in a health promotion context. Cost-effectiveness studies are also rare in the context of universal school-based health promotion; the results of this trial will provide a basis for decision-makers in the field.

If online *StresSOS* proves efficacious and non-inferior to face-to-face delivery, it will have far-reaching implications for health promotion for C&A, both in and outside the school setting. Embedded in a nation-wide network of health professionals experienced in school-based prevention, *StresSOS* could be easily implemented into the routine, and thus would potentially have a significant impact on mental health promotion in C&A.

### Trial status

Recruitment of the participants has not yet begun. The recruitment period for the trial will start in October 2018 with the school-based baseline assessment within the ProHEAD Consortium and last until March 2020.

## Additional file

Additional file 1: SPIRIT 2013 checklist: recommended items to address in a clinical trial protocol and related documents. (DOC 121 kb)

## Abbreviations

AHSQ: Actual Help-Seeking Questionnaire
AUDIT: Alcohol Use Disorders Identification Test
BASH-B: Barriers to Adolescents Seeking Help Scale
C&A: Children and adolescents
CEAC: Cost-effectiveness acceptability curve
CRAFFT-d: Car, Relax, Alone, Forget, Friends, Trouble
DRKS: German Clinical Trials Register
DSMB: Data and safety monitoring board
DSM-IV: Diagnostic and Statistical Manual of Mental Disorders, fourth edition
GHSQ: General Help-Seeking Questionnaire
IASMHS: Inventory of Attitudes Toward Seeking Mental Health Services
ICER: Incremental cost-effectiveness ratio
ITT: Intention-to-treat
KIDSCREEN-10: Health-related quality of life measure for C&A
KKS: Coordination Center for Clinical Trials
MHL: Mental health literacy
MRV: Mannheimer Modul zum Ressourcenverbrauch
PHQ-A: Patient Health Questionnaire-9 modified for Adolescents
ProHEAD: Promoting Help-seeking using E-technology for ADolescents
QALY: Quality-adjusted life year
RCT: Randomized controlled trial
SAPAS: Standardized Assessment of Personality – Abbreviated Scale
SDQ: Strengths and Difficulties Questionnaire
SEED: Short Evaluation of Eating Disorders
SEKJ: Inventory of Self-Esteem for Children and Adolescents
SSKJ 3–8 R: Stress and Coping Questionnaire for Children and Adolescents
WCS: Weight Concerns Scale
WTP: Willingness-to-pay
